# Clinical Genetics of Polydactyly: An Updated Review

**DOI:** 10.3389/fgene.2018.00447

**Published:** 2018-11-06

**Authors:** Muhammad Umair, Farooq Ahmad, Muhammad Bilal, Wasim Ahmad, Majid Alfadhel

**Affiliations:** ^1^Department of Biochemistry, Faculty of Biological Sciences, Quaid-i-Azam University, Islamabad, Pakistan; ^2^Division of Genetics, Department of Pediatrics, King Abdulaziz Medical City, Ministry of National Guard-Health Affairs (NGHA), King Abdullah International Medical Research Centre, King Saud bin Abdulaziz University for Health Sciences, Riyadh, Saudi Arabia

**Keywords:** polydactyly, PPD, PAP, digit anomalies, limb defects, extra digits/toes

## Abstract

Polydactyly, also known as *hyperdactyly* or *hexadactyly* is the most common hereditary limb anomaly characterized by extra fingers or toes, with various associated morphologic phenotypes as part of a syndrome (syndromic polydactyly) or may occur as a separate event (non-syndromic polydactyly). Broadly, the non-syndromic polydactyly has been classified into three types, i.e.; preaxial polydactyly (radial), central polydactyly (axial), and postaxial polydactyly (ulnar). Mostly inherited as an autosomal dominant entity with variable penetrance and caused by defects that occur in the anterior-posterior patterning of limb development. In humans, to-date at least 10 loci and six genes causing non-syndromic polydactyly have been identified, including the *ZNF141*, *GLI3*, *MIPOL1*, *IQCE*, *PITX1*, and the *GLI1*. In the present review, clinical, genetic and molecular characterization of the polydactyly types has been presented including the recent genes and loci identified for non-syndromic polydactyly. This review provides an overview of the complex genetic mechanism underlie polydactyly and might help in genetic counseling and quick molecular diagnosis.

## Introduction

The term polydactyly, “poly means many and dactylos means digits” is acknowledged to the 17th century Kerchring (1988). Polydactyly or polydactylism refers to the occurrence of supernumerary digits, toes or any complex duplication of digital parts. This situation was described as “superfluous fingers” in the 16th century by Ambrose Parey (Bell, 1953). It is among the most common congenital limb anomaly observed immediately at birth, manifesting in a variety of forms, ranging from complete or incomplete duplication of digits. Its occurrence is estimated 1.6–10.7/1000 in general population, 0.3–3.6/1000 in live births and males are often affected twice as females (Mellin, 1963; Castilla et al., 1973). Phenotypically, polydactyly is an extremely heterogeneous deformity (Temtamy and McKusick, 1978), with high tendency for the involvement of right hand than the left, upper limbs are more affected than the lower and left foot more affected than the right (Castilla et al., 1973; Malik et al., 2014). Polydactyly occurs in both syndromic and non-syndromic forms. The two most common types of polydactyly are postaxial polydactyly (PAP) categorized by an extra digit at the fifth finger or toe and preaxial polydactyly (PPD), having a digit (superfluous) attached on the greater toe or thumb side (Winter and Tickle, 1993; Lange and Müller, 2017). While, the mesoaxial polydactyly is a very rare form of digit deformity involvingduplication of second, third, or fourth digits. The present review extends the classification presented by Malik (2014). Total 435 and 3267 entries were obtained using the mesh “polydactyly” in the OMIM and PubMed [NCBI]. They include both syndromic and non-syndromic postaxial, preaxial, and complex polydactylies. Non-syndromic polydactyly types have been presented in Supplementary Table S1, while syndromic polydactyly entries from OMIM have been summarized in Supplementary Table S2, which might be helpful for clinicians and researchers in proper diagnosis, surgery, and treatment of polydactyly cases.

## Polydactyly Classification

**FIGURE 1:**
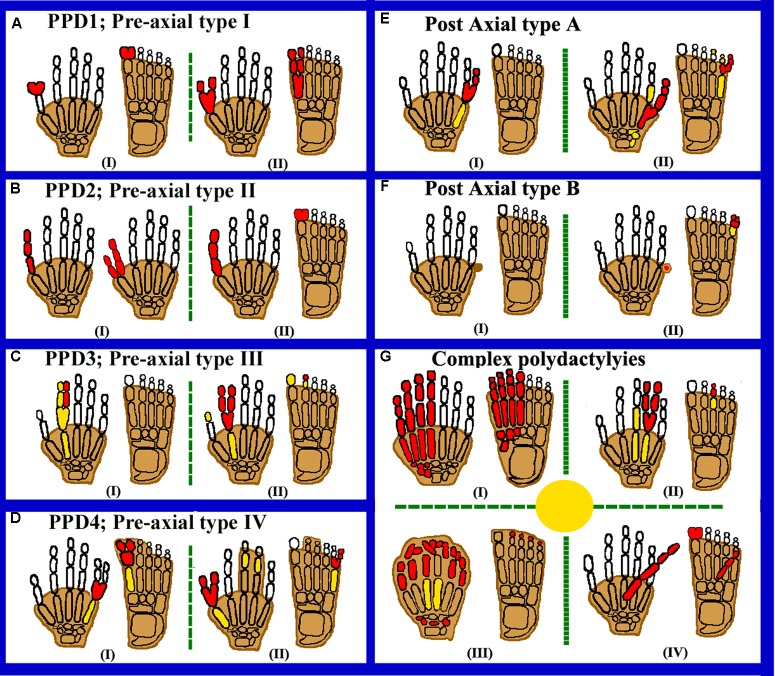
Cartoon diagrams of autopods showing preaxial, postaxial, and complex polydactylies. Red filled elements portray the affected/polydactylous digits. Yellow filled elements portray dysplastic/hypoplastic bones and shaded digits represent syndactyly. **(A)** Representing PPD1 including (I) bifid thumb, hallucal polydactyly and (II) duplication of thumb and hallux. **(B)** Representing PPD2 including (I) opposable triphalangeal thumb and (II) non-opposable triphalangeal thumb. **(C)** Representing PPD3 including (I,II) duplication of the second digit. **(D)** Representing PPD4 including (I) toe webbing (Cross-type I) and (II) finger/toe webbing (Cross type II). **(E)** Representing PAPA, having (I) well developed fifth digit and (II) more proximal branching of the (5^th^) fifth digit. **(F)** Representing PAPB, (I) Pedunculated postminimus and (II) bifid fifth toe-pedunculated postminimus. **(G)** Representing complex polydactylies (I) showing mirror image preaxial duplications. (II) Central polydactyly (mesoaxial) in hand and foot. (III) Haas type polydactyly with complete syndactyly. (IV) Palmer/dorsal polydactyly.

The polydactyly classification systems and basis of classification has been summarized in the Supplementary Table S3. Temtamy and McKusick (1978) scheme is the most widely used classification among different genetic counselors, radiologists, and clinicians. In this system, polydactyly has been classified into three types, i.e., PPD, PAP, and complex types (Figure 1).

## Preaxial Polydactyly (PPD)

Preaxial polydactyly refers to polydactyly where the additional digit grows toward the first digit of the hand (radial side; thumb) or foot (medially). The incidence of PPD is reported as high as 10/300 births (Deng et al., 2015; Manske et al., 2017). Wassel (1969) classified PPD into seven types that is very useful for hand surgeons, helping them to manage thumb duplication (Supplementary Figure S1). Later, four types of PPDs were classified by Temtamy and McKusick (1978) as polydactyly of thumb/hallux (type 1), triphalangeal thumb polydactyly (TPT, type 2), polydactyly of pointer finger (type 3), and crossed polydactyly or polysyndactyly (CP, type 4) (Figures 1A–D).

### Preaxial Polydactyly Type 1 (Thumb Polydactyly)

Thumb polydactyly is the most common type representing the duplication of a biphalangeal thumb (MIM 174400; Figure 1A). Usually observed as unilateral form, while in bilateral cases, hands are more preferentially affected and the left hand PPD are rare as compared to right hand PPD (Malik et al., 2014). There is a low occurrence of familial reappearance and high prevalence of affected males than females (Orioli and Castilla, 1999). Heptadactyly or hexadactyly is also a type of thumb polydactyly in which triplication of thumb occurs, resulting in seven digits (Zuidam et al., 2008). Familial PPD type 1 only shows autosomal dominant inheritance pattern with reduced penetrance (Castilla et al., 1973: Orioli and Castilla, 1999; Burger et al., 2018). Preaxial type 1 polydactyly is caused by sequence variants in the sonic hedgehog (SHH) enhancer, called zone of polarizing activity (ZPA) regulatory sequence (*ZRS*) (Lettice et al., 2002; Wieczorek et al., 2010; Perez-Lopez et al., 2018). During limb development, the pattern of the anterior-posterior (AP) axis is determined by the expression of *SHH* (MIM 600725) in a region called the zone of polarizing activity (ZPA). A *cis*-regulatory enhancer known as *ZRS* (MIM 605522); that controls the SHH limb expression. *ZRS* is almost 750–800 bp highly conserved functional element from humans to fish, located within intron 5 of the *LMBR1* (MIM 605522) gene. Recently, pathogenic variants in the 500 bp upstream of the ZRS (evolutionary conserved, non-coding region), termed the pre-ZRS (pZRS), have been associated with polydactyly phenotype (Potuijt et al., 2018).

Hallux polydactyly (MIM 601759) is known to exist as a predominant presentation or an isolated entity (Castilla et al., 1973). The incidence of hallux duplication is 2.4/100,000 as compared to thumb polydactyly incidence in South America, which is 1.65/10,000, respectively. In hallux duplication, females are less affected as compared to males, mostly unilateral and predominantly involved the right foot (Orioli and Castilla, 1999). Up to date the molecular basis of non-syndromic hallucal polydactyly remains unknown.

### Triphalangeal Thumb Polydactyly (TPT; Preaxial Polydactyly Type 2)

Preaxial polydactyly type 2 (MIM 174500), or TPT (replaced biphalangeal thumb) where the thumb has an extra middle phalanx with abnormally long and thin first metacarpal, having epiphyses at both ends (Figure 1B). TPT is usually symmetrical and bilateral (Swanson and Brown, 1962). TTP is inherited dominantly with incomplete penetrance (Temtamy and McKusick, 1978). Tsukurov et al. (1994) for the first time mapped PPD type 2 on chromosome 7q36. *ZRS* present within intron 5 of the *LMBR1* gene responsible for PPD type 2 pathogenesis (Lettice et al., 2003).

### Polydactyly of Index Fingers (Preaxial Polydactyly Type 3)

Preaxial polydactyly type 3 (MIM 174600) is very rare disorder segregating in an autosomal dominant fashion. In this type, the index finger is usually duplicated. One or two triphalangeal digits replace the thumb. The metacarpal of the accessory digit shows distal epiphysis, due to such phenotypes PPD type 3 is separated from PPD type 2 or TPT (Swanson and Brown, 1962; Temtamy and McKusick, 1978). The “central polydactyly” and “index polydactyly” are sometimes lumped together and considered as thumb duplication variants (Wood, 1970). The extra digit normally is in major angulations or radial deviation, and the normal digit may have deviated toward the ulnar side of varying degree (Graham and Ress, 1998; Perez-Lopez et al., 2018; Figure 1C).

### Polysyndactyly, CP (Preaxial Polydactyly Type 4)

In polysyndactyly (MIM 174700) the thumb is duplicated mildly, the distal phalanxes show radial deviation or with a broad and bifid thumb. Syndactyly of third and fourth fingers is rarely present (Temtamy and McKusick, 1978; Burger et al., 2018). In the feet, the first toe shows polydactyly, and the first metacarpal is tibially deviated and short. It is noted that this entity is different from synpolydactyly, in which syndactyly within the web is related with an additional digit (Malik, 2012). The word “CP” is typically used for the presence of postaxial and PPD, while the main change is observed in the additional digits axis of hands and feet (Figure 1D).

In CP type 1, PPD in feet and a PAP in hands is mostly observed. CP type 2 shows PAP of feet combined with PPD of hands (Temtamy and McKusick, 1978). Mutations in the *ZRS* and the *GLI3* genes have been reported for the appearance of CP type 1, which is also allelic to PAP A/B (Radhakrishna et al., 1999; Perez-Lopez et al., 2018). To-date 216 mutations have been reported in the *SHH* gene associated with different types of limb anomalies (HGMD, Stenson et al., 2017).

## Postaxial Polydactyly (PAP)

Postaxial polydactyly have one or more than one extra fibular or ulnar digit along the fifth finger (Figure 1E). Its prevalence as live births is 1–2/1000, with some ethnic group differences (Zhou et al., 2004). PAP is 75% more common than PPD, about 8% of bilateral PAP cases with lower and upper limbs are associated with many other congenital syndromic defects (Supplementary Table S2). Specifically for the PAP, two distinct categories have been recognized, i.e., postaxial type A, having extra digit (fully developed digit) and postaxial type B (incomplete digit), both these types differ in severity, penetrance estimates and inheritance pattern (Temtamy and McKusick, 1978; Watson and Hennrikus, 1997). PAP-type A is further classified into eight genetic types: PAP-type A1-A7 and PAP-type 8 with or without Ellis–van Creveld syndrome (EVC) phenotypes. In humans, six potential disease-causing genes have been reported to cause different types of non-syndromic polydactylies. This includes a GLI family zinc finger 3 gene (*GLI3*, MIM 165240), the IQ Domain-Containing Protein E (*IQCE*; MIM 617631), a zinc finger protein 141 gene (*ZNF141*, MIM 194648), a mirror image polydactyly gene (*MIPOL1*, MIM 606850), a paired-like homeodomain 1 gene (*PITX1*, MIM 602149), and GLI family zinc finger 1 gene (*GLI*; 165220) (Supplementary Table S1).

### Postaxial Polydactyly Type A

**FIGURE 2:**
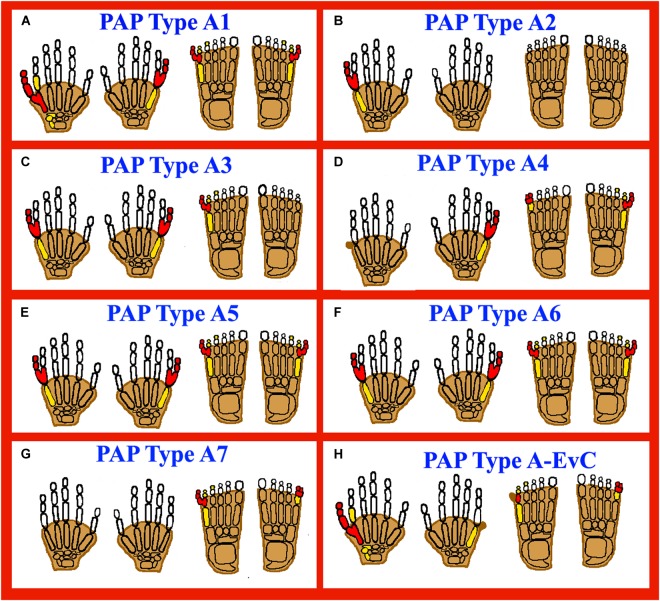
Cartoon diagrams of autopods showing postaxial polydactyly (PAP) types A (PAPA1-PAPA7 and PAPA type A-EVC). Red filled elements portray the affected/polydactylous digits, yellow filled elements portray dysplastic/hypoplastic bones and shaded digits represent syndactyly. **(A)** Representing PAPA1, having a well developed extra digit in both upper and lower limbs. **(B)** Representing PAPA2, having extra digit restricted to upper limbs. **(C)** PAPA3, showing well developed extra digits in both upper and lower limbs. **(D)** PAPA4, having a well developed extra digit, PAPB also reported (skin tag without bone). **(E,F)** PAPA5, 6: Well developed extra digits and toes. **(G)** Representing PAPA7, having extra digit restricted to only toes. **(H)** Representing PAP type A-EVC, having PAP type A and PAP type B in both upper and lower limbs.

In PAP type A, fully developed extra digit articulates with either a duplicated metatarsal or metacarpal or with the fifth metatarsal or metacarpal (Temtamy and McKusick, 1978). The duplicated digit may have one to three bony elements depending upon its size, which results in flexion wrinkle and a well-developed nail. Postaxial digit and the fifth ray shows a high variable angle of articulation (i.e., <30–180°). In most cases, the extra digit may cause problems in daily life (non-functional digit). PAP type A, mostly segregate as an autosomal dominant trait with reduced penetrance (Kucheria et al., 1981; Castilla et al., 1997). However, several Pakistani families having PAP segregating in an autosomal recessive inheritance have been identified (Umm-e-Kalsoom et al., 2012; Kalsoom et al., 2013; Palencia-Campos et al., 2017; Umair et al., 2017b) and can be classified into further eight types (Figure 2; Supplementary Table S1).

### Postaxial Polydactyly Type A1 (PAPA1)

PAPA1 and PAPB (MIM 174200) and PPD type IV (MIM 174700) are inherited in autosomal dominant fashion, caused by pathogenic heterozygous mutations in the *GLI3* gene (MIM 165240) located on chromosome 7p14.1.

Radhakrishna et al. (1997) reported a five generation family with fifteen affected individuals having PAPA phenotypes using genome-wide linkage analysis. A max LOD score of 4.21 was obtained using a marker D7S801 on chromosome 7p15–q11.23. Thus, a pathogenic heterozygous variant was identified in the *GLI3* gene (Radhakrishna et al., 1997). Similarly, Furniss et al. (2007) reported a patient having PAP type B in hands. Genetic analysis identified a disease causing heterozygous variant in the *GLI3* gene, predicted to result in premature termination and might causing nonsense-mediated mRNA decay (Furniss et al., 2007).

A three-generation family from Saudi Arabia was reported by Al-Qattan (2012) having phenotypes such as PAP of the hands, broad thumbs, and syndactyly (cutaneous) of the hands and feet. The genetic and molecular analysis identified a heterozygous 2-bp frameshift deletion in the *GLI3* gene predicted to cause truncation in the N-terminal part of the gene (Al-Qattan, 2012; Figure 2A). To-date 225 mutations have been identified in the *GLI3* gene causing different types of limbs anomalies including polydactyly phenotypes (HGMD; Stenson et al., 2017).

### Postaxial Polydactyly Type A2 (PAPA2)

Postaxial polydactyly type A2, having autosomal dominant inheritance (MIM 602085) maps on chromosome 13q21–q32, and the causative gene have not been identified. Linkage was performed in a family presenting PAPA features by Akarsu et al. (1997) and the family was excluded for the 7p15–q11.23 locus, known to cause PAPA1 (MIM 174200). The family showed linkage to chromosome 13q21–q32, having a max LOD score of 2.35 for the marker D13S1230 (Akarsu et al., 1997).

Furthermore, using standard karyotyping, van der Zwaag et al. (2010) identified heterozygous *de novo* inverted duplication in the long arm of chromosome 13, in a single affected boy with bilateral PAP of the hands. FISH analysis and array comparative genomic hybridization (aCGH) further confirmed the heterozygous duplication, respectively (van der Zwaag et al., 2010; Figure 2B).

### Postaxial Polydactyly Type A3 (PAPA3)

Postaxial polydactyly type A3 having an autosomal dominant inheritance (MIM 607324) maps on chromosome 19p13.2–p13.1. Zhao et al. (2002) identified a max LOD score of 5.85 for the marker D19S221, corresponding to a physical distance of about 2.5 Mb. The 16 affected individuals showed phenotypes such as well-developed and functional extra postaxial digits on hands and/or feet. Seven affected individuals had polydactyly of the hands, and no other phenotypes were observed (Figure 2C). The causative gene has not been identified yet.

### Postaxial Polydactyly Type A4 (PAPA4)

Postaxial polydactyly type A4 was first described in a six generation family with 11 affected individuals (Galjaard et al., 2003). In the affected individuals both PAPA and syndactyly phenotypes were variable in both the upper/lower limbs, with no other associated anomalies (Figure 2D). Genetic and molecular analysis using whole-genome screening identified maximum 2-point LOD score of 3.18 with marker D7S1799. PAPA4 shows autosomal dominant inheritance (MIM 608562), maps on chromosome 7q22.1 and the causative gene has not been identified.

### Postaxial Polydactyly Type A5 (PAPA5)

A Pakistani consanguineous family having autosomal recessive inheritance was asserted from a remote area showed features of PAP type A (bilateral) in both hands and feet. While no other abnormality was observed in the affected individuals and their normal parents (Umm-e-Kalsoom et al., 2012; Figure 2E). Using genome wide linkage analysis by typing highly polymorphic microsatellite markers Umm-e-Kalsoom et al. (2012) found linkage with max LOD score of 3.84 on chromosome 13q13.3–q21. PAPA5 (MIM 263450) was mapped to a 17.87cM region (D13S1288 and D13S632 markers) and the first evidence of autosomal recessive PAP mapped to chromosome 13q13.3–q21 was reported. The causative gene has not been identified.

### Postaxial Polydactyly Type A6 (PAPA6)

Kalsoom et al. (2013), while studying a Pakistani consanguineous family having a well-developed (bilateral) PAP in both hands and feet (Figure 2F), identified a 6.53 Mb linkage interval on chromosome 4p16.3–p16.2. Twenty-seven highly polymorphic microsatellite markers were typed and a maximum 3.38 multipoint LOD score was obtained at marker D4S412. Using techniques such as aCGH analysis and whole exome sequencing, Kalsoom et al. (2013) identified a missense mutation (p.Thr474Ile) in the *ZNF141* gene (MIM 194648) that segregated with the disease phenotype within the family and classified as PAPA6 (MIM 615226).

### Postaxial Polydactyly Type A7 (PAPA7)

Umair et al. (2017b) identified a homozygous splice site variant in the *IQCE* gene (MIM 617631), validated using WES and minigene assay in a consanguineous Pakistani family, having five affected individuals. Two affected members of the family were exome sequenced followed by Sanger sequencing to segregate the variant with disease phenotype within the family. PAPA7 (MIM 617642) is characterized by PAP restricted to lower limbs, with well-developed nails (Umair et al., 2017b; Figure 2G). The *IQCE* gene has 22 exons, encoding a 695 amino acid protein and located on chromosomal location 7p22.3. To-date only a single splice acceptor site variant (c.395-1G > A; p.Gly132Valfs^∗^22) have been reported in the *IQCE* gene associated with PAP restricted to lower limbs (Umair et al., 2017b). The protein product of *IQCE* binds to EFCAB7 forming a protein complex that acts as a positive mediator of hedgehog (Hh) signaling. The IQCE-EFCAB7 complex further interacts with a second protein module formed by EVC-EVC2 to tether it to the base of cilia (Pusapati et al., 2014). Mutations in either *EVC* or *EVC2* cause Ellis–van Creveld syndrome (EVC), a condition characterized by decreased Hh signaling and polydactyly as one of the phenotypes. Hence, pathogenic mutations in the *IQCE* have been proposed to cause polydactyly phenotypes involved in abnormal Hh signaling (Umair et al., 2017b).

### Postaxial Polydactyly Type A With or Without EVC Phenotypes (PAP Type A-EVC)

Palencia-Campos et al. (2017) recently reported homozygous loss of function variants in the *GLI1* gene (MIM 165220) located on chromosome 12q13.3 in three families (two from Turkey and one from Pakistan) with limb anomalies. Four affected individuals of Pakistani family showed isolated PAP features (Figure 2H). The two other families originating from Turkey have four affected individuals showing PAP associated with short stature, atrial septal defect (ASD), mild nail dysplasia, or genu valgum classified as typical EVC syndrome features. Interestingly, all patients with homozygous loss of function variants in *GLI1* had a common PAP phenotype. While, the family originating from Pakistan had isolated PAP feature and no other anomaly was observed. The *GLI1* gene (MIM 165220; NM_005269.2) has 12 exons, encodes an 1106 amino acids protein and located on chromosomal 12q13.3. To-date only three loss of function mutations (c.2340G > A; p.Trp780, c.1930C > T; p.Gln644^∗^, c.337C > T; p.Arg113^∗^) have been reported in the *GLI1* gene causing PAP and EVC phenotypes (Palencia-Campos et al., 2017). The author concluded that variability of the phenotype spectrum of the GLI1 patients might be associated with the position of the mutation and dosage effect. GLI family of proteins (GLI1, GLI2, and GLI3) are transcriptions factors of Hh signaling pathway, and have been reported to be involved in cell proliferation and patterning during embryonic development (Vortkamp et al., 1991).

### Postaxial Polydactyly Type B

In PAP type B, the richest type of polydactyly in a variety of populations, the extra digit may not be well developed and thus occurs in the form of a skin, from a negligible sign of small protuberance to spine-like outgrowth on the ulnar side of fifth finger, or a nubbin-like 2–3 cm long “pedunculated postminimus,” usually having a nail (Castilla et al., 1973; Figure 1F). The articulation site of the fifth digit along this nubbin is variable and frequently through as a small cutaneous bridge. Preferentially, the left hand and the upper limbs are mostly affected. This type of polydactyly shows more complicated genetics and thus, the estimated penetrance is about 43% (Castilla et al., 1973; Holmes et al., 2018).

In literature several dominantly inherited loci have been associated with PAP including PAP1 located on 7p14.1 with the associated gene *GLI3* (MIM 174200), PAP2 located on chromosome 13q21–q32 (MIM 263450), and PAPA3 also having features of PAP-A/B with chromosomal address 19p13.1–13.2 (MIM 607324). A PAP locus with PAP-A/B phenotypes and partial cutaneous syndactyly was also reported by Galjaard et al. (2003) on chromosome 7q21–q34 (MIM 608562).

## Complex Types of Polydactyly

Complex polydactylies are categorized separately as they have a different phenotype than PAP or PPD (Figure 1G).

### Mirror-Image Polydactyly (MIP)

In MIP (MIM 135750), posterior digits duplication occurs, while the anterior digits are completely exchanged in reverse order by the posterior digits. Thus, the arrangement of an extra digit is in descending order from the central digit, e.g., little finger, ring finger, middle finger, index finger, and the middle finger, ring finger, little finger with thumb/hallux absent (Temtamy and McKusick, 1978; Figure 1G [I]). Martin et al. (1993) reported an individual with complete duplication of all fingers having nine digits on right and 10 digits on the left hand, thus having a bilaterally complicated configuration. Isolated MIP is very rare and inherited as an autosomal dominant entity, while it is usually described as a part of Laurin-Sandrow syndrome (MIM 135750). Mutation in *MIPOL1* gene (MIM 606850) cause one of the phenotypes of MIP localized at chromosome 14q13 (Kondoh et al., 2002). *PITX1* gene (MIM 602149) mutations have also been reported to causes MIP with lower limb malformations (Klopocki et al., 2012). To-date 17 mutations have been reported in the *PITX1* gene causing different types of limbs anomalies (HGMD; Stenson et al., 2017).

### Mesoaxial or Central Polydactyly

Mesoaxial or Central polydactyly is a “hidden” duplications with apparent syndactyly or in the middle part of the hand, synonychia, may be present as a mass of tissue, though all mesoaxial types are not hidden (Figure 1G [II]). Central polydactyly like second finger duplication is dominantly inherited (Graham and Ress, 1998). The deformity is usually bilateral, and the fourth digit is duplicated most frequently; these duplications are more common than the index digit duplication (Temtamy and McKusick, 1978).

### Palmer and Dorsal Polydactyly

Palmer polydactyly is a very rare disorder in which extra digit usually arise from the ventrum or dorsum part of autopods. It may be shown as a poorly developed digit ray or a small skin (tag), or a developed digit with or without nail and implanted into the autopod as a hook (Figure 1G [III]). The mobile duplicated digit in the hand and a ventral or palmer origin digit was also reported (Nair et al., 2001). Similarly, Hussain et al. (2007) described duplicated digit originating from the dorsum of the foot.

### Haas Type Polysyndactyly

In Haas type polysyndactyly (MIM 186200), all the digits are fused cutaneously, and there is a postaxial or a preaxial extra ray in the web (Haas, 1940). Due to a complete syndactyly, the movement of digits is restricted and the fusion of adjacent fingers gives a cup-shaped appearance to the hand (Figure 1G [IV]). Malik (2012) generally classified Haas type polydactyly as type 5 syndactyly. On the basis of evidence, it has been observed that Haas type polysyndactyly is genetically heterogeneous. Mutations in the *ZRS* and the *GLI3* are known to cause Haas type polysyndactyly (Wieczorek et al., 2010; Lohan et al., 2014).

## Polydactyly Associated Pathways

The current genetic and molecular classification suggest that at least six different types of polydactylies are caused by mutations in two different genes, i.e., SHH enhancer *ZRS* and the *GLI3*, causing PPD1, PPD2, TPT-PS, PPD4, PAPA1, and Haas type (Supplementary Table S1). Thus, the important genetic role of these two factors and also the intimate SHH signaling pathway between GLI3 and SHH during limb development cannot be ignored. In humans, between 4 and 8 weeks of gestation, the process of limb embryogenesis starts resulting in the growth of the limb bud from lateral plate mesoderm. Different types of signal centers develop in the growing limb, guiding the mechanism of anterior/posterior patterning, i.e., determination of digit number and morphogenesis (Talamillo et al., 2005). Dysregulation of the Sonic hedgehog–Patched–Gli (SHH–Ptch–Gli) pathway leads to several human diseases, including birth defects, skeletal anomalies and cancers. The SHH–Gli3-activated Ptch transcription pathway elements are highly conserved and are very important in controlling the AP limb patterning. The main key players in the SHH–Gli3 pathway include the Smo, SHH, Gli3, IQCE, Ptch1 along with retinoic acid and the bone morphogenetic protein (BMP) that is also essential for AP patterning.

Ptch1 and Smo are also intermediate genes in the SHH pathway, Smo activates SHH signaling in the presence of SHH. While, in the absence of SHH the Ptch receptor inhibits Smo function thus having an inhibitory function. The most important gene in this pathway is the *GLI3* and pathogenic mutations in the *GLI3* cause both pre- and post-axial polydactyly phenotypes. The Gli3 exists in two different forms (Gli3R and Gli3A). The Gli3R exist as a repressor and converted into an activated Gli3A by the Smo when the SHH–Ptch interactions take place. The proportions of the Gli3R/Gli3 are directly involved in the development of digit types and numbering (Masuya et al., 2007; Park et al., 2008). Yet, the exact genotype and phenotype correlation with Gli3 mutations have not been developed due to the complex interaction of several other genes in the pathogenesis of polydactyly and bifunctional transcriptional switch (Maas and Fallon, 2004; Maas et al., 2011).

The involvement of these key players (GLI3 or ZRS) in the development of limbs phenotypes and their role in the signaling pathway is very important. However, the complexity of the polydactyly disorder lies in the phenomenon of genetic heterogeneity. As there is a broad spectrum of polydactyly types caused due to pathogenic sequence variants in the coding sequence of the *GLI3* gene or within the 800 bp *ZRS* region, thus genetic heterogeneity is expected across different ethnic groups and populations (Al-Qattan, 2018). Detail *ZRS* molecular mechanism underline pre-axial polydactyly pathogenesis has been described in Supplementary Figure S2. Many genetic factors involved in the etiology of syndromic and non-syndromic polydactyly/limb malformations still remain to be identified. Classification of the newly identified genes is the need of the day, which might help the clinicians and researchers in getting a clear idea of molecular pathogenesis and result in quick genetic diagnosis.

## Conclusion and Perspectives

Advances in human genetics have revealed several new isolated and syndromic polydactyly types that enhanced our knowledge and understanding of several genes responsible for genetic limbs pathogenesis. The genetics of polydactyly is a highly complex and not only restricted to Mendelian inheritance. Mechanisms such as genetic and allelic heterogeneity, epigenetic factors, associated genes, the role of enhancers/suppressors, and different type of environmental and developmental factors plays a very important role. Numerous detailed genetic, epidemiological, molecular, and embryological studies of polydactyly had observed substantial variations in the phenotype, prevalence, transmission, expressivity, and suggesting a high etiological of phenotypic heterogeneity (Castilla et al., 1973; Pérez-Molina et al., 1993; Talamillo et al., 2005; Lange and Müller, 2017). In addition, several other factors including variable expressivity, incomplete penetrance, difference in genetic background, environmental influence and epigenetic phenomena probably played roles in developing differential phenotypes in human (Umair et al., 2017b). In the past, numerous authors feeling the need for an organized overview, have attempted to devise a classification system for polydactyly. Different polydactyly classifications their advantages and back draws has been presented in Supplementary Table S4.

With the advent of new technologies, such RNA sequencing, systematic bioinformatics, next-generation sequencing (NGS) new genes and disease-causing mutations will be discovered that might help in establishing genotype–phenotype correlations in the near future. RNA-Seq can analyze the unceasingly changing transcriptome, post-transcriptional modifications, alternative spliced transcripts, SNPs/pathogenic variants, gene fusion, and gene expression over time. In the recent years, WGS/WES (NGS) has tremendously accelerated the diagnosis of familial and sporadic cases providing quick, accurate and cost effective genetic screening (Umair et al., 2017a, 2018). Proper genotype-phenotype correlations might help in future genetic testing, control medical impediments, enhance our knowledge about newly identified diseases and their associated genes thus providing substantial knowledge about large number of variants identified through NGS technologies, which in recent years might be used for screening and molecular diagnosis. Furthermore, the effects of specific pathogenic variant in causal gene might cause the concerned phenotype, while different variants in modifier genes might explain the phenotypic variability in several cases. Co-relating the large scale sequencing data with correct phenotypic information might help to develop predictive phenotypic models using the latest clustered regulatory interspaced short palindromic repeats/CRISPR associated protein 9 (CRISPR–Cas9) technologies might contribute substantially to the understanding of limb developmental pathways, help to clarify the pathogenesis of polydactyly and carry on experimental treatment study. These strategies might identify highest-yield molecular targets for therapeutic interventions, which still remain a challenge for future research.

## Author Contributions

MU wrote the article. FA and MB collected data and rephrased the article. WA and MA supervised and approved the article.

## Conflict of Interest Statement

The authors declare that the research was conducted in the absence of any commercial or financial relationships that could be construed as a potential conflict of interest.

## Supplementary Material

FIGURE S1 The Wassel-preaxial classification.

FIGURE S2 Schematic representation of the ZRS molecular mechanism. **(A)** Showing different sites and functional regions within the ZRS. The ZRS is represented by an orange rectangle, the WMS 5 bp site (red), the Ebox (green), and Hox sites (gray). The positions of the five ETS sites (transcription factors) that control the position of the expression boundary are represented by the pink circles. The green box contributes to regulating levels. The two ETV binding sites are shown below the ZRS rectangle that controls posterior restriction. **(B)** Schematic representation of interaction summary between the HOXD genes clusters and the ZRS to strengthen the SHH expression. The ZRS (orange box) and the SHH (purple), and the HOXD cluster, including the early enhancer and the late enhancer are shown in blue boxes.

TABLE S1 Non-syndromic polydactyly types, genes.

TABLE S2 Syndromic polydactyly disorders, genes.

TABLE S3 Polydactyly classification systems and basis of classification.

TABLE S4 Merits and demerits of different classification systems.
