# Supplementary figures and images for: Clinical Genetics of Polydactyly: An Updated Review
Source: Front Genet. 2018 Nov 6;9:447. doi: 10.3389/fgene.2018.00447 (PMC6232527; doi:10.3389/fgene.2018.00447)

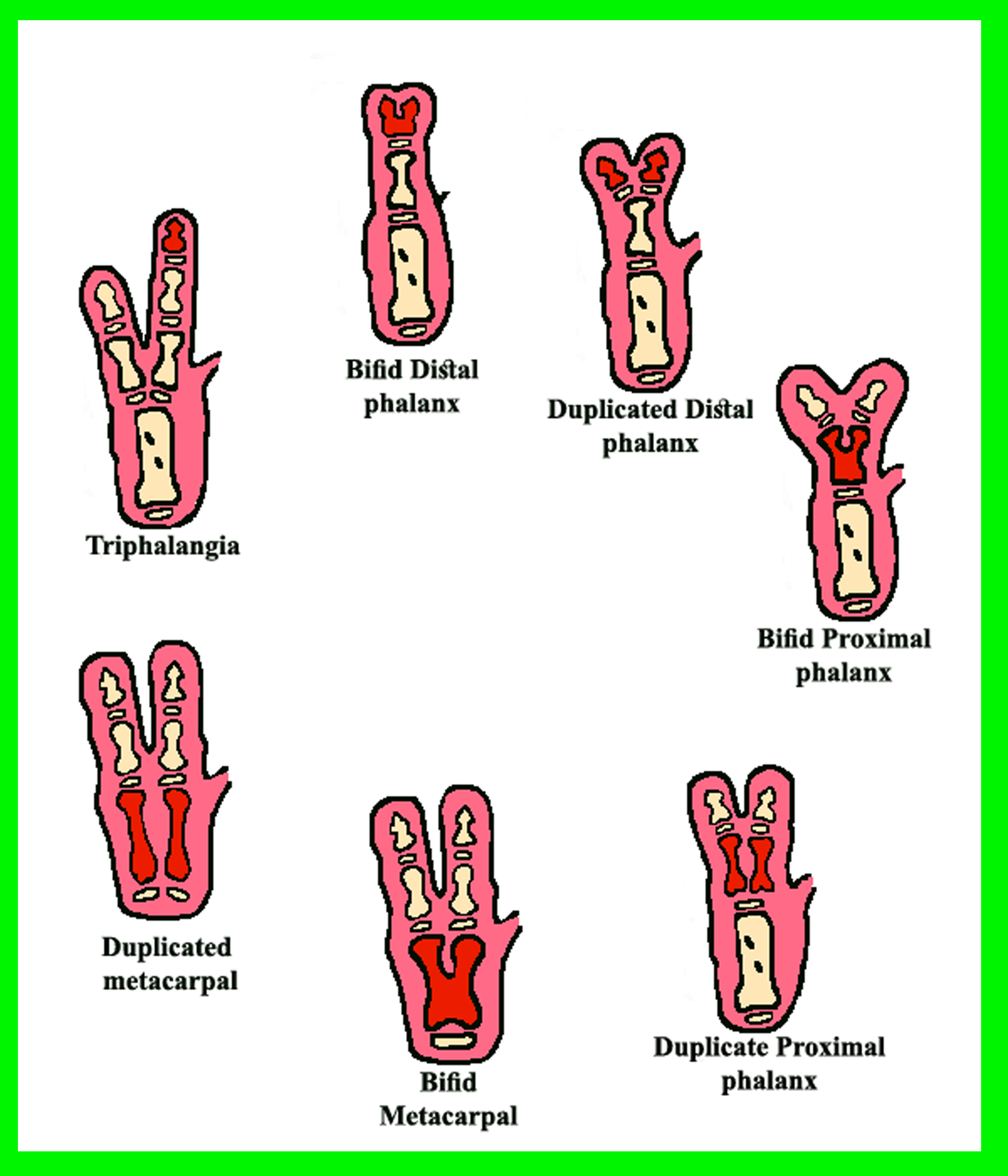

Supplement: FIGURE S1 — The Wassel-preaxial classification. [file Image_1.TIF]

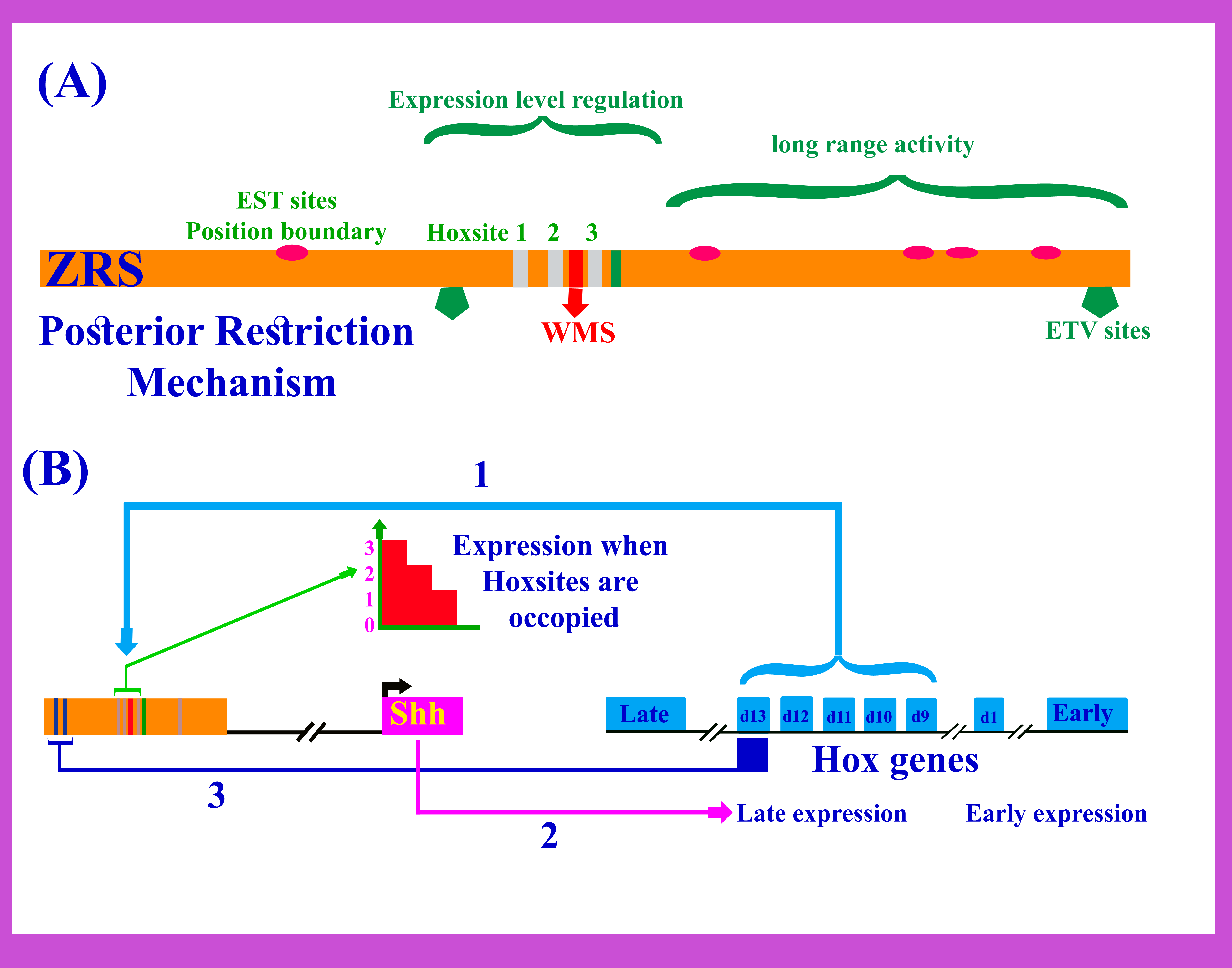

Supplement: FIGURE S2 — Schematic representation of the ZRS molecular mechanism. (A) Showing different sites and functional regions within the ZRS. The ZRS is represented by an orange rectangle, the WMS 5 bp site (red), the Ebox (green), and Hox sites (gray). The positions of the five ETS sites (transcription factors) that control the position of the expression boundary are represented by the pink circles. The green box contributes to regulating levels. The two ETV binding sites are shown below the ZRS rectangle that controls posterior restriction. (B) Schematic representation of interaction summary between the HOXD genes clusters and the ZRS to strengthen the SHH expression. The ZRS (orange box) and the SHH (purple), and the HOXD cluster, including the early enhancer and the late enhancer are shown in blue boxes. [file Image_2.TIF]
