# Supplementary material for: Clinical Genetics of Polydactyly: An Updated Review
Source: Front Genet. 2018 Nov 6;9:447. doi: 10.3389/fgene.2018.00447 (PMC6232527; doi:10.3389/fgene.2018.00447)
Supplement: TABLE S1 — Non-syndromic polydactyly types, genes. [file Table_1.DOCX]

**Supplementary Table 1:** Characterization of disease causing non-syndromic polydactyly types

| **Genes** | **Disease** | **Inheritance** | **Locus** | **OMIM** |
| --- | --- | --- | --- | --- |
| ***U*** | PPD1 | AD | U | 174400 |
| ***ZRS/SHH*** | PPD2 | AD | 7q36 | 174500 |
| ***U*** | PPD3 | AD | U | 174600 |
| ***GLI3*** | PPD IV | AD | 7p14.1 | 174700 |
| ***U*** | HALLUX TYPE | U | 2q31.1-31.2 | 601759 |
| ***GLI3*** | PAPA1 | AD | 7p14.1 | 174200 |
| ***U*** | PAPA2 | AD | 13q21-q32 | 602085 |
| ***U*** | PAPA3 | AD | 19p13.1-p13.2 | 607324 |
| ***U*** | PAPA4 | AD | 7q21-q34 | 608562 |
| ***U*** | PAPA5 | AR | 13q13.3-q21.2 | 263450 |
| ***ZNF141*** | PAPA6 | AR | 4p16.3 | 615226 |
| ***IQCE*** | PAPA7 | AR | 7p22.3 | 617642 |
| ***GLI1*** | PAP with or without EvC phenotypes | AR | 12q13.3 | 165220 |
| ***MIPOL1*** | COMPLEX TYPE; MIP | AD | 14.11.2-13 | 606850 |
| ***PITX1*** | MIRROR IMAGE POLYDACTYLY | AD | 5q31.1 | 602149 |
| ***ZRS/SHH*** | HAAS TYPE | AD | 7q36 | 186200 |

*Abbreviations: AR, autosomal recessive; AD, autosomal dominant; U, unknown*
