# Supplementary material for: Clinical Genetics of Polydactyly: An Updated Review
Source: Front Genet. 2018 Nov 6;9:447. doi: 10.3389/fgene.2018.00447 (PMC6232527; doi:10.3389/fgene.2018.00447)
Supplement: TABLE S2 — Syndromic polydactyly disorders, genes. [file Table_2.DOCX]

**Supplementary Table (S2):** Syndromic polydactyly disorders, genes and OMIM number

| **Gene** | **Disease** | **Inheritance** | **Locus** | **OMIM** | **Type** |
| --- | --- | --- | --- | --- | --- |
| **ALX3** | FRONTONASAL DYSPLASIA | AR | 1p13.3 | 136760 | Syndromic |
| **DDX59** | OROFACIODIGITAL SYNDROME V; OFD5 | AR | 1q32.1 | 174300 | Syndromic |
| **UBE2T** | FANCONI ANEMIA, COMPLEMENTATION GROUP T; FANCT | AR | 1q32.1 | 616435 | Syndromic |
| **LBR** | GREENBERG DYSPLASIA; GRBGD | AR | 1q42.12 | 215140 | Syndromic |
| **SDCCAG8** | BARDET-BIEDL SYNDROME 16; BBS16 | AR | 1q43 | 615993 | Syndromic |
| **CKAP2L** | FILIPPI SYNDROME; FLPIS | AR | 2q13 | 272440 | Syndromic |
| **RNU4ATAC** | MICROCEPHALIC OSTEODYSPLASTIC PRIMORDIAL DWARFISM, TYPE I; MOPD1 | AR | 2q14.2 | 210710 | Syndromic |
| **WDPCP** | BARDET-BIEDL SYNDROME 15; BBS15 | AR | 2p15 | 615992 | Syndromic |
| **WDPCP** | CONGENITAL HEART DEFECTS, HAMARTOMAS OF TONGUE, AND POLYSYNDACTYLY; CHDTHP | AR | 2p15 | 217085 | Syndromic |
| **IFT172** | SHORT-RIB THORACIC DYSPLASIA 10 WITH OR WITHOUT POLYDACTYLY; SRTD10 | AR | 2p23.3 | 615630 | Syndromic |
| **TTC21B** | SHORT-RIB THORACIC DYSPLASIA 4 WITH OR WITHOUT POLYDACTYLY ; SRTD4 | AR | 2q24.3 | 613819 | Syndromic |
| **TMEM237** | JOUBERT SYNDROME 14; JBTS14 | AR | 2q33.1 | 614424 | Syndromic |
| **BBS5** | BARDET-BIEDL SYNDROME 5; BBS5 | AR | 2q31.1 | 615983 | Syndromic |
| **ARL6** | BARDET-BIEDL SYNDROME 3; BBS3 | AR | 3q11.2 | 600151 | Syndromic |
| **CD96** | C SYNDROME | AR | 3q13.1-q13.2 | 605039 | Syndromic |
| **LZTFL1** | BARDET-BIEDL SYNDROME 17; BBS17 | AR | 3p21.31 | 615994 | Syndromic |
| **NPHP3** | MECKEL SYNDROME, TYPE 7; MKS7 | AR | 3q22.1 | 267010 | Syndromic |
| **NPHP3** | RENAL-HEPATIC-PANCREATIC DYSPLASIA | AR | 3q22.1 | 208540 | Syndromic |
| **WNT7A** | ULNA AND FIBULA, ABSENCE OF, WITH SEVERE LIMB DEFICIENCY | AR | 3p25.1 | 276820 | Syndromic |
| **WNT7A** | FUHRMANN SYNDROME | AR | 3p25.1 | 228930 | Syndromic |
| **WNT7A** | ULNA AND FIBULA, ABSENCE OF, WITH SEVERE LIMB DEFICIENCY | AR | 3p25.1 | 276820 | Syndromic |
| **WDR19** | SHORT-RIB THORACIC DYSPLASIA 5 WITH OR WITHOUT POLYDACTYLY ; SRTD5 | AR | 4p14 | 614376 | Syndromic |
| **CC2D2A** | COACH SYNDROME | AR | 4p15.32 | 216360 | Syndromic |
| **CC2D2A** | MECKEL SYNDROME, TYPE 6; MKS6 | AR | 4p15.32 | 612284 | Syndromic |
| **EVC, EVC2** | ELLIS-VAN CREVELD SYNDROME | AR | 4p16.2 | 225500 | Syndromic |
| **BBS7** | BARDET-BIEDL SYNDROME 7; BBS7 | AR | 4q27 | 615984 | Syndromic |
| **NEK1** | SHORT-RIB THORACIC DYSPLASIA 6 WITH OR WITHOUT POLYDACTYLY;SRTD6 | AR | 4q33 | 263520 | Syndromic |
| **C5orf42** | JOUBERT SYNDROME 17; JBTS17 | AR | 5p13.2 | 614615 | Syndromic |
| **C5orf42** | OROFACIODIGITAL SYNDROME VI; OFD6 | AR | 5p13.2 | 277170 | Syndromic |
| **CEP120** | SHORT-RIB THORACIC DYSPLASIA 13 WITH OR WITHOUT POLYDACTYLY;SRTD13 | AR | 5q23.2 | 616300 | Syndromic |
| **RAB23** | CARPENTER SYNDROME TYPE 1 | AR | 6p11.2 | 201000 | Syndromic |
| **ICK** | ENDOCRINE-CEREBROSTEODYSPLASIA | AR | 6p12.2-p12.1 | 612651 | Syndromic |
| **UQCC2** | MITOCHONDRIAL COMPLEX III DEFICIENCY, NUCLEAR TYPE 7; MC3DN7 | AR | 6p21.31 | 615824 | Syndromic |
| **PTHB1** | BARDET-BIEDL SYNDROME 9; BBS9 | AR | 7p14.3 | 615986 | Syndromic |
| **CEP41** | JOUBERT SYNDROME 15; JBTS15 | AR | 7q32.2 | 614464 | Syndromic |
| **WDR60** | SHORT-RIB THORACIC DYSPLASIA 8 WITH OR WITHOUT POLYDACTYLY; SRTD8 | AR | 7q36.3 | 615503 | Syndromic |
| **CSPP1** | JOUBERT SYNDROME 21; JBTS21 | AR | 8q13.1-q13.2 | 615636 | Syndromic |
| **GDF6** | LEBER CONGENITAL AMAUROSIS 17; LCA17 | AR | 8q22.1 | 615360 | Syndromic |
| **C8orf37** | CONE-ROD DYSTROPHY 16; CORD16 | AR | 8q22.1 | 614500 | Syndromic |
| **DNAI1** | CILIARY DYSKINESIA, PRIMARY, 1, WITH OR WITHOUT SITUS INVERSUS | AR | 9p13.3 | 244400 | Syndromic |
| **TRIM32** | BARDET-BIEDL SYNDROME 11; BBS11 | AR | 9q33.1 | 615988 | Syndromic |
| **CRB2** | VENTRICULOMEGALY WITH CYSTIC KIDNEY DISEASE; VMCKD | AR | 9q33.3 | 219730 | Syndromic |
| **INPP5E** | JOUBERT SYNDROME 1 ;MOUSE POLYDACTYLY | AR | 9q34.3 | 213300 | Syndromic |
| **INPP5E** | JOUBERT SYNDROME 1; JBTS1 | AR | 9q34.3 | 213300 | Syndromic |
| **INPP5E** | MENTAL RETARDATION, TRUNCAL OBESITY RETINAL DYSTROPHY, AND MICROPENIS | AR | 9q34.3 | 610156 | Syndromic |
| **WDR34** | SHORT-RIB THORACIC DYSPLASIA 11 WITH OR WITHOUT POLYDACTYLY;SRTD11 | AR | 9q34.11 | 615633 | Syndromic |
| **TCTN3** | OROFACIODIGITAL SYNDROME IV; OFD4 | AR | 10q24.1 | 258860 | Syndromic |
| **TCTN3** | JOUBERT SYNDROME 18; JBTS18 | AR | 10q24.1 | 614815 | Syndromic |
| **BBIP1** | BARDET-BIEDL SYNDROME 18; BBS18 | AR | 10q25.2 | 615995 | Syndromic |
| **TMEM138** | JOUBERT SYNDROME 16 | AR | 11q12.2 | 614465 | Syndromic |
| **C2CD3** | OROFACIODIGITAL SYNDROME XIV; OFD14 | AR | 11q13.4 | 615,948 | Syndromic |
| **PHOX2A** | FIBROSIS OF EXTRAOCULAR MUSCLES, CONGENITAL, 2 | AR | 11q13.4 | 602753 | Syndromic |
| **DYNC2H1** | SHORT-RIB THORACIC DYSPLASIA 3 WITH OR WITHOUT POLYDACTYLY;SRTD3 | AR | 11q22.3 | 613091 | Syndromic |
| **CEP164** | NEPHRONOPHTHISIS 15 | AR | 11q23.3 | 614845 | Syndromic |
| **HYLS1** | HYDROLETHALUS SYNDROME 1 | AR | 11q24.2 | 236680 | Syndromic |
| **BBS10** | BARDET-BIEDL SYNDROME 10; BBS10 | AR | 12q21.2 | 615987 | Syndromic |
| **CEP290** | BARDET-BIEDL SYNDROME 14; BBS14 | AR | 12q21.32 | 615991 | Syndromic |
| **UBE3B** | KAUFMAN OCULOCEREBROFACIAL SYNDROME; KOS | AR | 12q24.11 | 244450 | Syndromic |
| **COG6** | CONGENITAL DISORDER OF GLYCOSYLATION, TYPE IIl; CDG2L | AR | 13q14.11 | 614576 | Syndromic |
| **KIAA0586** | SHORT-RIB THORACIC DYSPLASIA 14 WITH OR WITHOUT POLYDACTYLY;SRTD14 | AR | 14q23.1 | 616546 | Syndromic |
| **SMOC1** | MICROPHTHALMIA WITH LIMB ANOMALIES | AR | 14q24.2 | 206920 | Syndromic |
| **IFT43** | CRANIOECTODERMAL DYSPLASIA 3; CED3 | AR | 14q24.3 | 614099 | Syndromic |
| **TTC8** | BARDET-BIEDL SYNDROME 8; BBS8 | AR | 14q31.3 | 615985 | Syndromic |
| **ATD** | SHORT-RIB THORACIC DYSPLASIA 1 WITH OR WITHOUT POLYDACTYLY;SRTD1 | AR | 15q13 | 208500 | Syndromic |
| **BBS4** | BARDET-BIEDL SYNDROME 4; BBS4 | AR | 15q24.1 | 615982 | Syndromic |
| **STRA6** | MICROPHTHALMIA, SYNDROMIC 9 | AR | 15q24.1 | 601186 | Syndromic |
| **KIF7** | JOUBERT SYNDROME 12 | AR | 15q26.1 | 200990 | Syndromic |
| **KIF7** | ACROCALLOSAL SYNDROME; ACLS; HYDROLETHALUS SYNDROME 2; HLS2 | AR | 15q26.1 | 200990 | Syndromic |
| **BBS2** | BARDET-BIEDL SYNDROME 2; BBS2 | AR | 16q12.2 | 615981 | Syndromic |
| **IFT140** | SHORT-RIB THORACIC DYSPLASIA 9 WITH OR WITHOUT POLYDACTYLY; SRTD9 | AR | 16p13.3 | 266920 | Syndromic |
| **TMEM231** | MECKEL SYNDROME, TYPE 11; MKS11 | AR | 16q23.1 | 615397 | Syndromic |
| **TMEM231** | JOUBERT SYNDROME 20; JBTS20 | AR | 16q23.1 | 614970 | Syndromic |
| **B9D1** | MECKEL SYNDROME 9 | AR | 17p11.2 | 614209 | Syndromic |
| **MEOX1** | KLIPPEL-FEIL SYNDROME 2 | AR | 17q21.31 | 214300 | Syndromic |
| **ACOX1** | PREOXISOMAL-ACETYL Co-A OXIDASE DEFICIENTY | AR | 17q25.1 | 264470 | Syndromic |
| **RBBP8** | JAWAD SYNDROME; JWDS | AR | 18q11.2 | 251255 | Syndromic |
| **DLL3** | SPONDYLOCOSTAL DYSOSTOSIS- PRE AXIAL POLYDACTYLY | AR | 19q13.2 | 277300 | Syndromic |
| **B9D2** | MECKEL SYNDROME 10 | AR | 19q13.2 | 614175 | Syndromic |
| **MEGF8** | CARPENTER SYNDROME 2 | AR | 19q13.2 | 614976 | Syndromic |
| **DNMT3B** | IMMUNODEFICIENCY-CENTROMERIC INSTABILITY-FACIAL ANOMALIES SYNDROME 1; ICF1 | AR | 20q11.21 | 242860 | Syndromic |
| **PAX1** | PAIRED BOX GENE 1; PAX1;OTOFACIOCERVICAL SYNDROME 2 | AR | 20p11.22 | 167411 | Syndromic |
| **GDF5** | CHONDRODYSPLASIA, GREBE TYPE | AR | 20q11.22 | 200700 | Syndromic |
| **MKKS** | MCKUSICK-KAUFMAN SYNDROME | AR | 20p12.2 | 236700 | Syndromic |
| **MKKS** | BARDET-BIEDL SYNDROME 6; BBS6 | AR | 20p12.2 | 605231 | Syndromic |
| **HPE1** | HOLOPROSENCEPHALY-1 | AR | 21q22.3 | 236100 | Syndromic |
| **IFT27** | BARDET-BIEDL SYNDROME 19; BBS19 | AR | 22q12.3 | 615996 | Syndromic |
| **PIK3CA** | MEGALENCEPHALY-CAPILLARY MALFORMATION-POLYMICROGYRIA SYNDROME; MCAP | AR/AD | 3q26.32 | 602501 | Syndromic |
| **ZFP57** | ZINC FINGER PROTEIN 57, MOUSE, HOMOLOG OF; DIABETES MELLITUS, TRANSIENT NEONATAL, 1 | AR/AD | 6p22.1 | 612192 | Syndromic |
| **GDF6** | MICROPHTHALMIA, ISOLATED 4; MCOP4 | AR/AD | 8q22.1 | 613094 | Syndromic |
| **BMS1** | APLASIA CUTIS CONGENITA, NONSYNDROMIC; ACC | AR/AD | 10q11.21 | 107600 | Syndromic |
| **MIPOL1** | MIRROR-IMAGE POLYDACTYLY MIPOL1 | AR/AD | 14q13.3-q21.1 | 606850 | Syndromic |
| **DYRK1A** | MENTAL RETARDATION, AUTOSOMAL DOMINANT 7 | AR/AD | 21q22.13 | 614104 | Syndromic |
| **PHF8** | SIDERIUS X-LINKED MENTAL RETARDATION SYNDROME | XLR | Xp11.22 | 300263 | Syndromic |
| **PORCN** | FOCAL DERMAL HYPOPLASIA | XLD | Xp11.23 | 305600 | Syndromic |
| **EBP** | CHONRDODYSPLASIA PUNCTATA 2, X-LINKED DOMINANT | XLD | Xp11.23 | 601165 | Syndromic |
| **EBP** | MEND SYNDROME; MEND | XLR | Xp11.23 | 300960 | Syndromic |
| **RBM10** | TARP SYNDROME | XLR | Xp11.23 | 311900 | Syndromic |
| **EFNB1** | CRANIOFRONTONASAL DYSPLASIA; MOUSE POLYDACTYLY | XLD | Xq13.1 | 304110 | Syndromic |
| **MBTPS2** | IFAP SYNDROME WITH OR WITHOUT BRESHECK SYNDROME | XLR | Xp22.12-p22.11 | 308205 | Syndromic |
| **OFD1** | JOUBERT SYNDROME 10; JBTS10 | XLR | Xp22.2 | 300804 | Syndromic |
| **OFD1** | OROFACIODIGITAL SYNDROME I; OFDI | XLD | Xp22.2 | 311200 | Syndromic |
| **GPC3** | SIMPSPN-GOLABI-BEHMEL SYNDROME, TYPE I | XLR | Xq26.2 | 312870 | Syndromic |
| **PTCH2** | BASAL CELL NEVUS SYNDROME | AD | 1p34.1 | 109400 | Syndromic |
| **GLI2** | CULLER-JONES SYNDROME; CJS | AD | 2q14.2 | 615849 | Syndromic |
| **GLI2** | HOLPROSENCEPHALY 9 | AD | 2q14.2 | 610829 | Syndromic |
| **TP63** | ECTRODACTYLY, ECTODERMAL DYSPLASIA, AND CLEFT LIP/PALATE SYNDROME 3; EEC3 | AD | 3q28 | 604292 | Syndromic |
| **PROM1** | REINITIS PGMENTOSA 41 | AD | 4p15.32 | 612095 | Syndromic |
| **MSX1** | PRE-AXIAL POLYDACTYLY, CLEFT PLATE, HYPODONTIA, MICRODONTIA | AD | 4p16.2 | 142983 | Syndromic |
| **EVC** | WEYERS ACROFACIAL DYSOSTOSIS; WAD | AD | 4p16.2 | 193530 | Syndromic |
| **FGFR3** | THANATOPHORIC DYSPLASIA, TYPE I | AD | 4p16.3 | 187600 | Syndromic |
| **PITX1** | CLUBFOOT, CONGENITAL OR MIRROR-IMAGE POLYDACTYLY; CCF | AD | 5q31.1 | 119800 | Syndromic |
| **NSD1** | BECKWITH-WIEDEMANN SYNDROME | AD | 5q35.2-q35.3 | 606681 | Syndromic |
| **TFAP2B** | CHAR SYNDROME | AD | 6p12.3 | 169100 | Syndromic |
| **GJA1** | OCULO-DENTO-DIGITAL SYNDROME | AD | 6q22.31 | 164200 | Syndromic |
| **TFAP2A** | BRANCHIOOCULOFACIAL SYNDROME; BOFS | AD | 6p24.3 | 113620 | Syndromic |
| **EEC1** | EEC syndrome-1; ECTRODACTYLY, ECTODERMAL DYSPLASIA, AND CLEFT LIP/PALATE SYNDROME | AD | 7q11.2-q21.3 | 129900 | Syndromic |
| **GLI3** | PALLISTER-HALL SYNDROME; | AD | 7p14.1 | 146510 | Syndromic |
| **HOXA13** | PREAXIAL DEFICIENCY POSTAXIAL POLYDACTYLY, AND HYPOSPADIAS | AD | 7p15.2 | 176305 | Syndromic |
| **TWIST1** | ROBINOW-SORAUF SYNDROME | AD | 7p21.1 | 180750 | Syndromic |
| **P4HB** | COLE-CARPENTER SYNDROME 1 | AD | 17q25.3 | 112240 | Syndromic |
| **LMBR1** | SYNDACTYLY, TYPE IV; SDTY4 | AD | 7q36.3 | 186200 | Syndromic |
| **LMBR1** | LAURIN-SANDROW SYNDROME | AD | 7q36.3 | 135750 | Syndromic |
| **LMBR1** | TIBIA, ABSENCE OF, WITH POLYDACTYLY; THYP | AD | 7q36.3 | 188740 | Syndromic |
| **FGFR1** | PFEIFFER SYNDROME | AD | 8p11.23-p11.22 | 101600 | Syndromic |
| **PUF60** | VERHEIJ SYNDROME; VRJS | AD | 8q24.3 | 615583 | Syndromic |
| **PTCH1** | BASAL CELL NEVUS SYNDROME | AD | 9q22.32 | 109400 | Syndromic |
| **PAX2** | PAPILLORENAL SYNDROME | AD | 10q24.31 | 120330 | Syndromic |
| **SUFU** | BASAL CELL NEVUS SYNDROME | AD | 10q24.32 | 109400 | Syndromic |
| **FGFR2** | APERT SYNDROME | AD | 10q26.13 | 101200 | Syndromic |
| **FGFR2** | PFEIFFER SYNDROME | AD | 10q26.13 | 101600 | Syndromic |
| **COL2A1** | ACHONDROGENESIS, TYPE II | AD | 12q13.11 | 200610 | Syndromic |
| **TBX5** | HOLT-ORAM SYNDROME | AD | 12q24.21 | 142900 | Syndromic |
| **CDKN1C** | BECKWITH-WIEDEMANN SYNDROME | AD | 11p15.4 | 130650 | Syndromic |
| **ICR1** | BECKWITH-WIEDEMANN SYNDROME | AD | 11p15.5 | 616186 | Syndromic |
| **TBX3** | ULNAR-MAMMARY SYNDROME | AD | 12q24.21 | 181450 | Syndromic |
| **BMP4** | MICROPHTHALMIA, SYNDROMIC 6 | AD | 14q22.2 | 607932 | Syndromic |
| **SALL1** | TOWNES-BROCKS SYNDROME | AD | 16q12.1 | 107480 | Syndromic |
| **PKD1** | POLYCYSTIC KIDNEY DISEASE, ADULT TYPE I; APKD1 | AD | 16p13.3 | 173900 | Syndromic |
| **SALL4** | DUANE-RADIAL RAY SYNDROME | AD | 20q13.2 | 607323 | Syndromic |
| **WDR35** | SHORT-RIB THORACIC DYSPLASIA 7 WITH OR WITHOUT POLYDACTYLY; SRTD7 | AR | 2p24.1 | 614091 | Syndromic |
| **IFT80** | SHORT-RIB THORACIC DYSPLASIA 2 WITH OR WITHOUT POLYDACTYLY;SRTD2 | AR | 3q25.33 | 611263 | Syndromic |
| **FRAS1** | FRASER SYNDROME | AR | 4q21.21 | 219000 | Syndromic |
| **ROR2** | ROBINOW SYNDROME, AUTOSOMAL RECESSIVE; RRS | AR | 9q22.31 | 268310 | Syndromic |
| **BBS1** | BARDET-BIEDL SYNDROME 1; BBS1 | AR | 11q13.2 | 209900 | Syndromic |
| **DHCR7** | SMITH-LEMLI-OPITZ SYNDROME ;SLOS | AR | 11q13.4 | 270400 | Syndromic |
| **SC5DL** | LATHOSTEROLOSIS | AR | 11q23.3 | 607330 | Syndromic |
| **GRIP1** | FRASER SYNDROME | AR | 12q14.3 | 219000 | Syndromic |
| **FREM2** | FRASER SYNDROME | AR | 13q13.3 | 219000 | Syndromic |
| **MKS1** | DANDY-WALKER MALFORMATION WITH POSTAXIAL POLYDACTYLY; MECKEL SYNDROME 1 | AR | 17q22 | 249000 | Syndromic |
| **MKS1** | BARDET-BIEDL SYNDROME 13; BBS13 | AR | 17q22 | 615990 | Syndromic |
| **IHH** | INDIAN HEDGEHOG; IHH; ACROCAPITOFEMORAL DYSPLASIA; BRACHYDACTYLY, TYPE A1 | AR/AD | 2q35 | 600726 | Syndromic |
| **GLI3** | POLYDACTYLY, POSTAXIAL | AR/AD | 7p14.1 | 174200 | Syndromic |
| **TULP3** | TUBBY-LIKE PROTEIN 3; TULP3 | AR/AD | 12p13.33 | 604730 | Syndromic |
| **DACH1** | DACHSHUND, DROSOPHILA, HOMOLOG OF, 1; DACH1 | AR/AD | 13q21.33 | 603803 | Syndromic |
| **OTC** | ORNITHENE TRANS CARBIMYLASE DEFICIENCY | XLR | Xp11.4 | 311250 | Syndromic |
| **TBX22** | CLEFT PALATE WITH OR WITHOUT ANKYLOGLOSSIA, X-LINKED; CPX | XL | Xq21.1 | 303400 | Syndromic |
| **GLI3** | GREIG CEPHALOPOLYSYNDACTYLY SYNDROME | AD | 7p14.1 | 175700 | Syndromic |
| **LMBR1** | POLYDACTYLY, PREAXIAL II; PPD2 | AD | 7q36.3 | 174500 | Syndromic |
| **ALX4** | POLYDACTYLY MOUSE; PARIETAL FORAMINA 2; PFM2 | AD | 11p11.2 | 605420 | Syndromic |
| **H19** | BECKWITH-WIEDEMANN SYNDROME | AD | 11p15.5 | 103280 | Syndromic |
| **KCNQ1OT1** | BECKWITH-WIEDEMANN SYNDROME | AD | 11p15.5 | 604115 | Syndromic |
| **CCND2** | MEGALENCEPHALY-POLYMICROGYRIA-POLYDACTYLY-HYDROCEPHALUS SYNDROME 3 | AD | 12p13.32 | 615938 | Syndromic |
| **PIK3R2** | MEGALENCEPHALY POLYMICROGYRIA-POLYDACTYLY HYDROCEPHALUS SYNDROME | AD | 19p13.11 | 603387 | Syndromic |
| **FUZ** | NEURAL TUBE DEFECTS; FUZZY, DROSOPHILA, HOMOLOG OF; MOUSE POLYDACTYLY | AD | 19q13.33 | 610622 | Syndromic |
| **TBX1** | VELOCARDIOFACIAL SYNDROME | AD | 22q11.21 | 192430 | Syndromic |
| **U** | VATER/VACTERL ASSOCIATION | U |  | 192350 |  |
| **U** | TRIPHALANGEAL THUMB, NONOPPOSABLE | AD | U | 190600 | U |
| **U** | TRIPHALANGEAL THUMBS AND DISLOCATION OF PATELLA | AD | U | 190650 | U |
| **U** | TRIPHALANGEAL THUMBS WITH BRACHYECTRODACTYLY ULNA AND FIBULA, ABSENCE OF WITH SEVERE LIMB | AD | U | 190680 | U |
| **U** | SYNDACTYLY-POLYDACTYLY-EARLOBE SYNDROME; SPEL SYNDROME | AD | U | 186350 | U |
| **U** | POSTAXIAL OLIGODACTYLY, TETRAMELIC | AD | U | 176240 | U |
| **U** | POLYDACTYLY, POSTAXIAL WITH PROGRESSIVE MYOPIA | AD | U | 174310 | U |
| **U** | POLYDACTYLY, PREAXIAL I | AD | U | 174400 | U |
| **U** | NAGER ACROFACIAL DYSOSTOSIS | AD | U | 154400 | U |
| **U** | HALLUX VARUS AND PREAXIAL POLYSYNDACTYLY | AD | U | 234280 | U |
| **U** | DEAFNESS, CONGENITAL AND ONYCHODYSTROPHY | AD | U | 124480 | U |
| **U** | AASE SMITH SYNDROME TYPE I | AD | U | 147800 | U |
| **U** | COLOBOMAS-BRACHYDACTYLY (TYPE SORSBY) | AD | U | 601707 | U |
| **U** | POLYDACTYLY, PREAXIAL III | AD | U | 174600 | U |
| **U** | CEREBROOCULONASAL SYNDROME | AD | U | 605627 |  |
| **U** | BRACHYDACTYLY (PREAXIAL) HALLUX VARUS-THUMB ABDUCTION | AD | U | 112450 | U |
| **U** | BRACHYPHALANGY, POLYDACTYLY AND TIBIAL APLASIA/HYPOPLASIA | AD | U | 609945 | U |
| **U** | SPLIT-HAND/FOOT MALFORMATION 1; SHFM1 | AD | 7q21.2-q21.3 | 183600 | U |
| **U** | ARIMIA SYNDROME | AR | U | 243910 | U |
| **U** | POLYSYNDACTYLY WITH CARDIAC MALFORMATION | AR | U | 263630 | U |
| **U** | TIBIAL HEMIMELIA | AR | 8q24.1 | 275220 | U |
| **U** | TIBIA, ABSENCE OF HYPOPLASIA OF WITH POLYDACTYLY, RETROCEREBELLAR ARACHNOID CYST ETC | AR | U | 601027 | U |
| **U** | PSEUDOTRISOMY 13 SYNDROME | AR | U | 264480 | U |
| **U** | POLYSYNDACTYLY WITH CARDIAC MALFORMATION | AR | U | 263630 | U |
| **U** | POLYSYNDACTYLY, CROSSED | AR | U | 175690 | U |
| **U** | MOHR SYNDROME | AR | U | 252100 | U |
| **U** | MÜLLERIAN DERIVATIVES PERSISTENCE OF WITH LYMPHANGIECTASIA AND POSTAXIAL POLYDACTYLY | AR | U | 235255 | U |
| **U** | HEART-HAND-TYPE 4-WITH MESOAXIAL HEXADACTYLY | AR | U | 249670 | U |
| **U** | FACIOCARDIOMELIC SYNDROME | AR | U | 612731 | U |
| **U** | OLIVER SYNDROME | AR | U | 258200 | U |
| **U** | CEREBROFACIOTHORACIC DYSPLASIA | AR | U | 605627 | U |
| **U** | ACRO-CRANIO-FACIAL DYSOSTOSIS | AR | U | 201050 | U |
| **U** | CAMPTOBRACHYDACTYLY | AR | U | 114150 | U |
| **U** | OROFACIODIGITAL SYNDROME III; OFD3 | AR | U | 258865 | U |
| **U** | OROFACIODIGITAL SYNDROME IX; OFD9 | AR | U | 210350 | U |
| **U** | OROFACIODIGITAL SYNDROME X; OFD10 | AR | U | 263630 | U |
| **U** | BONNEAU SYNDROME | AR | U | 243910 | U |
| **U** | BIEMOND SYNDROME II | AR | U | 210350 | U |
| **U** | POLYDACTYLY, POSTAXIAL, TYPE A1; PAPA1 | U | 7p14.1 | 174200 | U |
| **U** | POLYDACTYLY, POSTAXIAL, TYPE A4 | U | 7q22 | 608562 | U |
| **U** | THAI SYMPHALANGISM SYNDROME | U | U | 608028 | U |
| **U** | SAKATI-NYHAN-ACROCEPHALOPOLYSYNDACTYLY TYPE III | U | U | 101120 | U |
| **U** | SCALP DEFECTS AND POSTAXIAL POLYDACTYLY | U | U | 181250 | U |
| **U** | POLYDACTYLY, POSTAXIAL WITH DENTAL AND VERTEBRAL ANOMALIES | U | U | 263540 | U |
| **U** | HYDROPS-ECTOPIC CALCIFICATION-MOTH-EATEN SKELETAL DYSPLASIA LBR | U | U | 215140 | U |
| **U** | HYPOMELIA WITH MÜLLERIAN DUCT ANOMALIES | U | U | 146160 | U |
| **U** | JOHNSON NEUROECTODERMAL SYNDROME | U | U | 147,770 | U |
| **U** | ACROCEPHALOPOLYSYNDACTYLOUS DYSPLASIA | U | U | 200995 | U |
| **U** | ACROCEPHALOSYNDACTYLY TYPE IV | U | U | 201020 | U |
| **U** | ACROFACIAL DYSOSTOSIS-TYPE RODRIGUEZ | U | U | 201170 | U |
| **U** | ACROFRONTOFACIONASAL DYSOSTOSIS TYPE 1 | U | U | 201180 | U |
| **U** | ACROFRONTOFACIONASAL DYSOSTOSIS TYPE 2 | U | U | 239710 | U |
| **U** | ACROMELIC FRONTONASAL DYSOSTOSIS | U | U | 603671 | U |
| **U** | ACROPECTORAL SYNDROME | U | 7q36 | 605967 | U |
| **U** | ACROPECTOROVERTEBRAL DYSPLASIA, F-FORM OF | U | U | 102510 | U |
| **U** | ACRORENAL SYNDROME | U | U | 102520 | U |
| **U** | ACRORENAL-MANDIBULAR SYNDROME | U | U | 200980 | U |
| **U** | AMINOPTERIN SYNDROME SINE AMINOPTERIN | U | U | 600325 | U |
| **U** | U MICROCEPHALY, CORPUS CALLOSUM DYSGENESIS, AND CLEFT LIP/PALATE | U | U | 601420 | U |
| **U** | LIVER FIBROCYSTIC DISEASE AND POLYDACTYLY | U | U | 605944 | U |
| **U** | HOLZGREVE SYNDROME | U | U | 236110 | U |
| **U** | MENTAL RETARDATION, MANDIBULOFACIAL DYSOSTOSIS, MICROCEPHALY, AND CLEFT PALATE | U | U | 610536 | U |
| **U** | FRYNS-AFTIMOS | U | U | 606155 | U |
| **U** | CRANIOFACIAL MALFORMATIONS, ASYMMETRIC WITH POLYSYNDACTYLY AND ABNORMAL SKIN | U | U | 304110 | U |
| **U** | CHONDRODYSPLASIA-SITUS INVERSUS-CYSTIC PANCREATIC DYSPLASIA | U | U | 603643 | U |
| **U** | FEMORAL-FACIAL SYNDROME | U | U | 134780 | U |
| **U** | ECTODERMAL DYSPLASIA SYNDROME WITH FACIAL SYNDROME AND PREAXIAL POLYDACTYLY OF FEET | U | U | 129540 | U |
| **U** | CRANIOFACIAL MALFORMATIONS, ASYMMETRIC WITH POLYSYNDACTYLY AND ABNORMAL SKIN | U | U | 601707 | U |
| **U** | OROFACIODIGITAL SYNDROME VII; OFD7 | U | U | 608,518 | U |
| **U** | OROFACIODIGITAL SYNDROME XI; OFD11 | U | U | 612913 | U |
| **U** | CUTIS MARMORATA TELANGIECTASIA CONGENITA | U | U | 219250 | U |
| **U** | GOITER, MULTINODULAR, CYSTIC RENAL DISEASE, AND DIGITAL ANOMALIES | U | U | 138790 | U |
| **U** | PALLISTER-KILLIAN SYNDROME ;HEXASOMY 12P, MOSAIC, INCLUDED | SOMATIC | 12p | 601803 | U |
| **U** | OROFACIODIGITAL SYNDROME VIII; OFD8 | XLR |  | 300,484 | U |
| **U** | ADAMSBAUM (1991) SYNDROME | U | U | WINTER-BARAITSER DYSMORPHOLOGY DATABASE | |
| **U** | ANANDAN (2008) STERNAL DEFECTS – APLASIA CUTIS CONGENITA-POLYDACTYLY | U | U | WINTER-BARAITSER DYSMORPHOLOGY DATABASE | |
| **U** | ARENS (1991) ACROFACIAL DYSOSTOSIS | U | U | WINTER-BARAITSER DYSMORPHOLOGY DATABASE | |
| **U** | NEURAL TUBE DEFECT-PREAXIAL POLYDACTYLY-VERTEBRAL ANOMALIES | U | U | WINTER-BARAITSER DYSMORPHOLOGY DATABASE | |
| **U** | BRUNONI (1984) RADIAL APLASIA; SHORT STATURE; UNUSUAL FACE | U | U | WINTER-BARAITSER DYSMORPHOLOGY DATABASE | |
| **U** | BRADDOCK (2003) LARYNGEAL WEBS; CHD; VERTEBRAL DEFECTS | U | U | WINTER-BARAITSER DYSMORPHOLOGY DATABASE | |
| **U** | BITOUN (1994)-GLAUCOMA-THUMB ANOMALIES-JOINT DISLOCATIONS | U | U | WINTER-BARAITSER DYSMORPHOLOGY DATABASE | |
| **U** | MORAVA (2004) FOCAL SKIN DEFECT-MICROPHTHALMIA-LIMB DEFECTS | U | U | WINTER-BARAITSER DYSMORPHOLOGY DATABASE | |
| **U** | CEREBRO-FRONTO-FACIAL SYNDROME TYPE III | U | U | WINTER-BARAITSER DYSMORPHOLOGY DATABASE | |
| **U** | COH SYNDROME-CRANIOSYNOSTOSIS; BIFID THUMB; MICROPENIS | U | U | WINTER-BARAITSER DYSMORPHOLOGY DATABASE | |
| **U** | FRYNS-LAGAE-RIZZO-POLYDACTYLY-GROWTH RETARDATION-SPASTICITY-UROGENITAL GANDHI (2008) | U | U | WINTER-BARAITSER DYSMORPHOLOGY DATABASE | |
| **U** | GARRETT-TRIPP-MR; POLYDACTYLY; HAIR ABSENCE; DERMATITIS; PERTHE’S DISEASE | U | U | WINTER-BARAITSER DYSMORPHOLOGY DATABASE | |
| **U** | GIANT DIENCEPHALIC HAMARTOMA-FACIAL CLEFT-EAR AND EYE ANOMALIES | U | U | WINTER-BARAITSER DYSMORPHOLOGY DATABASE | |
| **U** | HAMEED (1999) ACROCRANIOFACIAL SYNDROME | U | U | WINTER-BARAITSER DYSMORPHOLOGY DATABASE | |
| **U** | HAPPLE-TINSCHERT SYNDROME | U | U | WINTER-BARAITSER DYSMORPHOLOGY DATABASE | |
| **U** | HARTSFIELD (1984) HOLOPROSENCEPHALY; ECTRODACTYLY; CLEFT FACE | U | U | WINTER-BARAITSER DYSMORPHOLOGY DATABASE | |
| **U** | GÜL (2000)-CRANIOFACIAL ANOMALIES | U | U | WINTER-BARAITSER DYSMORPHOLOGY DATABASE | |
| **U** | GUSCHMANN (2001) MESOMELIC CAMPOMELIA-POLYDACTYLY-DANDY-WALKER | AR | U | WINTER-BARAITSER DYSMORPHOLOGY DATABASE | |
| **U** | HUANG (1999) HIRSCHSPRUNG, CONGENITAL HEART DEFECT, LARYNGEAL ANOMALIES | AR | U | WINTER-BARAITSER DYSMORPHOLOGY DATABASE | |
| **U** | GOOSSENS (2006) CONGENITAL HEART DISEASE-POLYDACTYLY-ECTOPIC NEUROPITUITARY | AR | U | WINTER-BARAITSER DYSMORPHOLOGY DATABASE | |
| **U** | MORTON (1998) LETHAL SKELETAL DYSPLASIA-ECTOPIC DIGITS | AR | U | WINTER-BARAITSER DYSMORPHOLOGY DATABASE | |
| **U** | BLAIR (2000) AUTOSOMAL RECESSIVE CRANIOSYNOSTOSIS SYNDROME | AR | U | WINTER-BARAITSER DYSMORPHOLOGY DATABASE | |
| **U** | BRAUN (1962) NEPHROSIS; DEAFNESS; BRACHYTELEPHALANGY | AR | U | WINTER-BARAITSER DYSMORPHOLOGY DATABASE | |
| **U** | ENCEPHALOCELE-RADIAL, CARDIAC, GASTROINTESTINAL, ANAL/RENAL ANOMALIES | AR | U | WINTER-BARAITSER DYSMORPHOLOGY DATABASE | |

AR=Autosomal Recessive; AD=Autosomal Dominant; U=Unknown; XL=X-linked
