# Supplementary material for: Clinical Genetics of Polydactyly: An Updated Review
Source: Front Genet. 2018 Nov 6;9:447. doi: 10.3389/fgene.2018.00447 (PMC6232527; doi:10.3389/fgene.2018.00447)
Supplement: TABLE S3 — Polydactyly classification systems and basis of classification. [file Table_3.DOCX]

**Supplementary Table 3:** Polydactyly classification systems and basis of classification.

| **Polydactyly Classification Systems** | 1. **Physical (topographic), morphological and anatomical systems.** |
| --- | --- |
|  | 1. **Physical (topographic) and focusing on inheritance patterns and phenotypes within family.** |
|  | 1. **Molecular and embryological approaches.** |
| **Basis of polydactyly classification** | 1. **The presence of polydactyly in the hands and/or in feet.** |
|  | 1. **Location of extra digit.** |
|  | 1. **Complete or partial duplication of extra digit.** |
|  | 1. **Presence of just a skin tag or bony element in the extra digit.** |
|  | 1. **Hereditary in nature or sporadic cause.** |
|  | 1. **Pattern of segregation within the family.** |
