# Supplementary material for: Clinical Genetics of Polydactyly: An Updated Review
Source: Front Genet. 2018 Nov 6;9:447. doi: 10.3389/fgene.2018.00447 (PMC6232527; doi:10.3389/fgene.2018.00447)
Supplement: TABLE S4 — Merits and demerits of different classification systems. [file Table_4.DOCX]

**Supplementary Table 4**: Merits and demerits of different classification systems (selected publications).

| **References** | **Merits** | **Demerits** |
| --- | --- | --- |
| Wassel, 1969 | Anatomical system; increasing severity/grading of thumb polydactyly; level of bifurcation | Restricted to radial polydactyly |
| Egawa, 1969 | Anatomical variations in digit rays | Restricted to treatment foot polydactyly |
| Nathan and Keniston, 1975 | Combined pre- and post-axial | Lacking Molecular and genetic characterization |
| Venn-Watson, 1976 | Morphological classification of metatarsals | Restricted to foot polydactyly |
| Miura, 1976 | Anatomical variations | Restricted to Triphalangeal thumb (pre-axial polydactyly) |
| Wood, 1978 | Anatomical; extensions in Wassel types (1969) | Restricted to hand polydactyly with no molecular characterization |
| Temtamy and McKusick, 1978 | Presentation in large families, inheritance pattern | Genetic characterization and surgical treatment missing. Restricted to hand polydactyly |
| Phelps and Grogan, 1985 | Morphological classification | Restricted to foot polydactyly |
| Ogino et al. 1988 | Anatomical; Extensions in Wassel types (1969) | Restricted to thumb polydactyly (pre-axial) |
| von Schrader, 1991 | Combined surgical approach with Temtamy-McKusick classification (1978) | Restricted to hand polydactyly types |
| Watanabe et al. 1992 | Morphological, type of ray involved, level of duplication; familial occurrence, and associations | Restricted to polydactyly of toes. |
| Light, 1992 | Developmental approach and treatment options; accounts for the absence of ossification centers of digital elements in infants | Restricted to treatment of preaxial polydactyly. Molecular characterization not explained. |
| Winter and Tickle, 1993 | Embryological; patterning and secondary limb modeling during development | Restricted to single gene mutations. Surgical characterization for surgeons not given. |
| Castilla et al. 1996 | Epidemiological and genetic basis | Surgical and molecular characterization |
| Rayan and Frey, 2001 | Increasing severity of little finger polydactyly (from distal to proximal axis) | Restricted to only Ulnar polydactyly |
| Lee and Park, 2006 | Characterization based on morphological and radiographical and operative findings | Restricted to only PAP in foot. |
| Al-Qattan et al. 2008 | Anatomical; size and severity of extra digit | Restricted to **ulnar polydactyly** |
| Zuidam et al. 2008 | Further extension of Wassel types (1969) | Restricted to radial polydactyly only (triphalangeal thumb and triplication). Molecular characterization and inheritance pattern restriction |
| Guo et al. 2013 | Based on Wassel classification | Extension of Wassel classification, with description of few other syndromic polydactyly |
| Seok et al. 2013 | SAM system (Syndactylism, Axis deviation, Metatarsal extent of extra digit). It summarized polydactyly on the basis of radiological and morphological criteria. It also customizes surgical methods and preoperatively predicts outcomes such as residual deformities. | Restricted with reference to genetic and molecular characterization |
| Malik et al. 2014 | Classification of both PED and PAP based on cardinal features, phenotypic variability and molecular advances. | Surgical treatment limitations |
| Duran et al. 2015 | Classification of ulnar polydactyly | Limited to ulnar polydactyly |
| Wall et al. 2016 | Synpolydactyly of the hand explanation | Limited to Synpolydactyly |
| Dijkman et al. 2016 | Clinical Presentation, Surgical Treatment, and Outcome in Radial Polydactyly | Restricted to Radial Polydactyly |
| Burger et al. 2016 | Describes complex osseous and soft tissue elements might be beneficial for the radial hand disorders and surgical treatments. | While, it drawbacks include complexity and the lack of visibility of certain anomalous components on radiography at a young age. |
| Evanson et al. 2016 | Radial Polydactyly classification | Restricted to Radial Polydactyly |
| Manske et al. 2017 | Extension of Wassel Classification | Limited to pre-axial polydactyly |

**References (Separate references for the Supplementary Table 4):**

**Al-Qattan MM, Al-Shanawani B, Al-Thunayan A, Al-Namla A. The clinical features of ulnar polydactyly in middle eastern population. J Hand Surg 2008: 33E (1): 47-52.**

Castilla EE, da Fonseca RL, Dutra MG, Bermejo E, Cuevas L, Martinex-Frias M-L. Epidemiological analysis of rare polydactylies. Am J Med Genet 1996; 65: 295-303.

Egawa T, Dor T, Yoshioka Y, Hiroshima K. Polydactyly of the foot. Jap J Plast Reconstr Surg 1969: 12: 363-368.

Lee HS, Park SS. Classification of postaxial polydactyly of foot. Foot Ankle Int 2006: 27 (5): 356-362.

Light TR. Treatment of preaxial polydactyly. Hand Clin 1992: 8: 161-175.

Miura T. Triphalangeal thumb. Plast Reconstr Surg 1976: 58: 587-594.

Nathan P, Keniston R. Crossed polydactyly. J Bone Joint Surg (Am) 1975: 57: 847-848.

Ogino T, Ishii S, Minami M. Radially deviated type of thumb polydactyly. J Hand Surg 1988: 13B: 315-319.

Phelps DA, Grogan DP. Polydactyly of foot. J Pediatr Orthop 1985: 5: 446-451.

Rayan GM, Frey B. Ulnar polydactyly. Plastic and Reconstructive Surg 2001: 107: 1449-1454.

Venn-Watson E. Problems in polydactyly of the foot. Orthop Clin North Am 1976: 7: 909-927.

von Schrader M. Polydaktylie der Hande. Vorschlage zu einer erweiterten Klassifikation. Handchir Mikrochir Plast Chir 1991: 23: 115-127.

Watanabe H, Fujitaw S, Oka I. Polydactyly of foot: an analysis of 265 cases and a morphological classification. Plast Reconstr Surg 1992: 89: 856-877.

Wood VE. Polydactyly and the triphalangeal thumb. J Hand Surg 1978: 3: 436-444.

Zuidam JM, Selles RW, Ananta M, Runia J, Hovius SER. A classification system f radial polydactyly: inclusion of triphalangeal thumb and triplication. J Hand Surg 2008: 33A: 373-377

Seok HH, Park JU, Kwon ST. New classification of polydactyly of the foot on the basis of syndactylism, axis deviation, and metatarsal extent of extra digit. Arch Plast Surg 2013; 40(3):232-237.

Burger EB, Hovius SE, Burger BJ, van Nieuwenhoven CA. The Rotterdam Foot Classification: A Classification System for Medial Polydactyly of the Foot. J Bone Joint Surg Am. 2016;98(15):1298-306. doi: 10.2106/JBJS.15.01416.

Manske MC, Kennedy CD, Huang JI. Classifications in Brief: The Wassel Classification for Radial Polydactyly. Clin Orthop Relat Res. 2017;475(6):1740-1746.

Synpolydactyly of the hand: a radiographic classification.

Wall LB, Bae DS, Oishi SN, Calfee RP, Goldfarb CA. J Hand Surg Eur Vol. 2016 ;41(3):301-7. doi: 10.1177/1753193415598281. Epub 2015 Aug 12.

Dijkman RR, van Nieuwenhoven CA, Hovius SE, Hülsemann W. Clinical Presentation, Surgical Treatment, and Outcome in Radial Polydactyly. Handchir Mikrochir Plast Chir 2016 ;48(1):10-17.

Duran A, Ciloglu NS, Buyukdogan H. A classification system for ulnar polydactyly and clinical series. J Hand Surg Am 2015; 40(5):914-921.

Evanson BJ, Hosseinzadeh P, Riley SA, Burgess RC. Radial Polydactyly and the Incidence of Reoperation Using A New Classification System. J Pediatr Orthop 2016;36(2):158-60.

Guo B, Lee SK, Paksima N. Polydactyly: a review. Bull Hosp Jt Dis (2013). 2013;71(1):17-23. Review

Malik S. Polydactyly: phenotypes, genetics and classification. Clin Genet 2014;85(3):203-12.
