# Supplementary material for: Clinical Genetics of Polydactyly: An Updated Review
Source: Front Genet. 2018 Nov 6;9:447. doi: 10.3389/fgene.2018.00447 (PMC6232527; doi:10.3389/fgene.2018.00447)
Supplement: Supplementary file 7 [file Data_Sheet_1.docx]

**Supplementary Figure 2: Schematic representation of the ZRS molecular mechanism.**

(A) Showing different sites and functional regions within the ZRS. The ZRS is represented by an orange rectangle, the WMS 5bp site (red), the Ebox (green), and Hoxsites (grey). The positions of the 5 ETS sites (transcription factors) that control the position of the expression boundary are represented by the pink circles. The green box contributes to regulating levels. The two ETV binding sites are shown below the ZRS rectangle that controls posterior restriction. (B) Schematic representation of interaction summary between the HOXD genes clusters and the ZRS to strengthen the SHH expression. The ZRS (orange box) and the SHH (purple), and the HOXD cluster, including the early enhancer and the late enhancer are shown in blue boxes.

**Summary**

Point mutations within the ZRS, act in a dominant fashion that causes digital abnormalities such as ‘‘The ZRS-associated syndromes’’. These include triphalangeal thumb-polysyndactyly (TTP), preaxial polydactyly type 2 (PPD2), Werner mesomelic syndrome (WMS) and syndactyly type 4 (SD4).

Several binding sites within the ZRS play different roles in the pattern of SHH expression in the embryonic limb bud. Including multiple binding sites for the ETS factors (ETS1/GABPa), that determines the boundary of SHH expression. Mutations resulting in an extra ETS site generation in the human ZRS results in an ectopic expression within the limb bud and extension of the expression boundary, thus act as dominant gain-of-activity which result in preaxial polydactyly phenotypes (Lettice et al., 2012, 2017; Laurell et al., 2012; Supplementary figure 2A).

While WMS is caused by point mutations within the 5-bp site or deletion encompassing this region. These point mutations result in loss of binding of a repressor and, an overall loss of functional activity. Thus, the ZRS have both gain and loss of activity and both these events result in dominant genetic effects on the phenotype.

Now, the early-expressing 50 HOXD proteins bind Hoxsites 1–3 (110bp) within the ZRS to establish SHH expression level in the initial limb development stages (Supplementary figure 2B, arrow 1). The levels of SHH expression depend on the number of Hox sites occupied. SHH, in turn, is crucial for the shift in HOXD gene expression to the later genes, e.g. HOXD13 (Supplementary figure 2B, arrow 2). The 50 HOXD genes, which include HOXD 9–13, are fundamental to limb patterning and with the HOXD9 gene expressing earliest in the limb bud, followed by HOXD13 being expressed late (Tarchini and Duboule, 2006). The early HOXD proteins (HOXD9–11) bind, suggesting a key role in establishing the activity levels of the ZRS. Loss of a HOX binding site counterbalances the increased and ectopic expression generated by the loss of the WMS repressor site. Thus, multiple HOXD factors coordinate, through binding at multiple sites, controlling the expression levels of SHH. HOXD13 subsequently binds to sites at the 5’ end of the ZRS, to maintain SHH expression later in limb development (Leal and Cohn, 2016; Lettice et al., 2017; Supplementary figure 2B, arrow 3).

Thus, there are two types of molecular etiology in the ZRS mutations that exhibit PPD. One type upregulates the SHH expression in the anterior limb buds through the loss of binding of a potential repressor (as a result of a point mutation), whereas the other type generates an extra ETS site that results in the extension of this expression boundary, thus resulting in preaxial polydactyly (Lettice et al., 2012, 2017; Laurell et al., 2012).

Recently, several studies have revealed that genetic alterations in locations other than the ZRS throughout the 1Mb encompassing SHH-LMBR1 could be associated with TPT phenotypes. A 2-kilobase (kb) deletion in a gene desert 240 kb upstream of SHH has been linked to familial TPT and hypertrichosis. Additionally, variations in the pre-ZRS (pZRS), a noncoding conserved region approximately 700 base pairs (bp) upstream to the ZRS, were reported in sporadic cases of preaxial polydactyly in humans (Petit et al, 2011; Xiang e al., 2017).

The genetic network of transcription factors and signaling pathways that converge at the ZRS is highly complex and needs further research studies.

**References for Supplementary Figure 2: not mentioned in the main text**

Laurell T, Vandermeer JE, Wenger AM, Grigelioniene G, Nordenskjöld A, Arner M, et al. A novel 13 base pair insertion in the sonic hedgehog ZRS limb enhancer (ZRS/LMBR1) causes preaxial polydactyly with triphalangeal thumb. Hum Mutat 33: 1063–1066 (2012).

Leal F, Cohn MJ. Loss and Re-emergence of Legs in Snakes by Modular Evolution of Sonic hedgehog and HOXD Enhancers. Curr Biol 26: 2966–2973 (2016).

Lettice LA, Devenney P, De Angelis C, Hill RE. The Conserved Sonic Hedgehog Limb Enhancer Consists of Discrete Functional Elements that Regulate Precise Spatial Expression. Cell Rep 20(6):1396-1408 (2017).

Lettice LA, Williamson I, Wiltshire JH, Peluso S, Devenney PS, Hill AE. Opposing functions of the ETS factor family define Shh spatial expression in limb buds and underlie polydactyly. Dev Cell 22: 459–467 (2012).

Petit F, Jourdain AS, Holder-Espinasse M, Keren B, Andrieux J, Duterque-Coquillaud M, et al. The disruption of a novel limb cis-regulatory element of SHH is associated with autosomal dominant preaxial polydactyly-hypertrichosis. Eur J Hum Genet 2016:24: 37–43 (2016).

Tarchini B, Duboule D. Control of Hoxd genes’ collinearity during early limb development. Dev Cell 10: 93–103 (2006).

Xiang Y, Jiang L, Wang B, Xu Y, Cai H, Fu Q. Mutational screening of GLI3, SHH, preZRS, and ZRS in 102 Chinese children with nonsyndromic polydactyly. Dev Dyn 246:392–402 (2017).
